# On Gall Stones

**Published:** 1864-04

**Authors:** J. L. W. Thudichum


### ON GALL STONES.

By Dr. J. L. W. THUDICHUM.

A knotty point which Dr. Thudichum has helped to solve is the nature of the morbid process to which gall-stones owe their origin. In this process two things have to be considered.

(1.) The decomposition of the bile, and consequent precipitation from it of the different materials which form the nuclei of gall-stones. (2.) The mode in which such precipitates come to form concretions. The cause of the decomposition—that is to say, the pathological condition of the liver in which it originates—is still a mystery. Dr. Thudichum suspects it to be some putrid ferment generated in, and absorbed from, the intestinal canal. But, be the cause what it may, the fact that the first step in the formation of a gall-stone is decomposition of the bile and precipitation of certain of its ingredients, is now undisputed; and by the light of the chemical changes which he has ascertained to take place in putrefying bile outside the body, the author attempts to explain the exact nature and successive steps of this decomposition. His explanation is too long for quotation. We would merely remark that it appears to us very reasonable, being (as he fairly claims it to be) "derived from all the data on record, with only so much of hypothesis as seemed justified by the amount of incontrovertible knowledge." As to the second point, viz., how concretion of the precipitates is effected, he shows the untenability of the old hypothesis, which ascribed it to inspissation of mucus or bile, and maintains that "the binding material of the nucleus of gall-stones is cholic acid, or chloidic acid, or both."

The author's description of the symptoms of gall-stone colic is of great value, because based exclusively upon cases in which the gall-stones were found after death. The general opinion that these concretions produce symptoms only when lodged in the *common* duct, has (he observes) of late "become very doubtful, as it has been found that calculi in the *hepatic* duct or ducts produce symptoms of a peculiar kind, differing from the symptoms of gall-stones in the gall-bladder and common duct by the absence of pain, end having the greatest resemblance to chronic intermittent fever. In one case treated by Frerichs for a long time by means of quinine and other febrifuge remedies, the rigors and other symptoms simulating fever were caused solely by calculi in the hepatic duct; their presence had not been suspected during the life of the patient."

*Retardation of the pulse*, he holds to be a pathognomonic symptom in this disease. In forty-five cases observed by Wolff, "the symptom of diminished frequency was so constantly found during frequent observations, that in doubtful cases (*i.e.*, in absence of jaundice) it was mainly relied upon for the diagnosis."

*Jaundice*, he believes to be by no means of such frequent occurrence during the passage of gall-stones as is commonly supposed. It was present in only twenty out of Wolff's forty-five cases. "The number of cases in which no jaundice appears is probably still larger in comparison to those in which it appears, as cases of the former kind easily escape diagnosis, while those accompanied by jaundice are never lost sight of."

Dr. Thudichum's remarks on the treatment of gall-stone disease, though advancing nothing positively new, contain some useful practical hints. He illustrates the necessity of leaving remedies, and directions for using them, with persons who have once had serious gall stone colic, so that, in the event of any sudden return, they may be employed without loss of time. The only known internal remedies effectual in mitigating the pain during the paroxysms are anodynes, such as opium, belladonna, and ether; but much caution, he shows, is needed in their administration, because when they have been taken in large doses they are apt, after cessation of the pain, to produce dangerous symptoms of narcotism. Both reason and experience, in his opinion, contra-indicate any abstraction of blood, either general or local, during the passage of the concretions. Their solution in the living body he believes to be impossible; their expulsion during the use of certain mineral waters (Carlsbad, Marienbad, Ems, Vichy, and others) he admits, but confesses himself unable to explain. He summarily condemns the practice of giving mercury in cases of jaundice from calculous obstruction, "or in any other cases of jaundice from whatever cause, syphilis excepted. Calomel is not a cholagogue, but diminishes the secretion of bile....." We cannot go the same length as Dr. Thudichum in condemnation of this remedy. To give it in jaundice from obstruction is manifestly senseless and mischievous. But, be the action of mercury what it may, in many cases of functional jaundice (especially in children) we find a moderate dose of calomel set the patient to rights so promptly, efficiently, and safely, that even Dr. Thudichum's strong disapproval will not persuade us to discard its use.—*From Dr. Thudichum's recent work on "Gall-Stones; their Chemistry, Pathology, and Treatment."* —*Med. Times and Gazette, Aug.* 8, 1863, *p.* 151.

